# Electrospun Nanofibers and Electrochemical Techniques for the Detection of Heavy Metal Ions

**DOI:** 10.3390/ma14113000

**Published:** 2021-06-01

**Authors:** Angela Malara, Antonio Fotia, Emilia Paone, Giulia Serrano

**Affiliations:** 1 Department of Civil, Energy, Environment and Material Engineering, Mediterranea University of Reggio Calabria, Via Graziella Loc Feo di Vito, 89124 Reggio Calabria, Italy; emilia.paone@unirc.it; 2 Consorzio Interuniversitario per la Scienza e la Tecnologia dei Materiali (INSTM), 50121 Firenze, Italy; giulia.serrano@unifi.it; 3 Department of Information Engineering, Infrastructures and Sustainable Energy, Mediterranea University of Reggio Calabria, Via Graziella Loc Feo di Vito, 89124 Reggio Calabria, Italy; antonio.fotia@unirc.it; 4 Department of Industrial Engineering—DIEF, University of Florence, Via di S. Marta 3, 50139 Firenze, Italy

**Keywords:** electrospinning, nanofibers, electrochemical techniques, heavy metals

## Abstract

Contamination by heavy metals is currently one of the most environmental concerns especially due to the toxicity, pervasiveness, and persistence of these substances. As they are not biodegradable, heavy metals are harmful not only for water, air, and soil but also for human health, even in very low traces. There is therefore a pressing need to develop an efficient, economic, and rapid analysis method to be applied in a wide range of conditions and able to detect very low contaminants concentrations. Currently, the most novel solution in this field is represented by the combination of electrospun nanofibers and highly sensitive electrochemical techniques. It has been proved that nanofibers, due to their outstanding properties, perfectly fit as sensing material when trace concentrations of heavy metals were investigated by anodic stripping voltammetry, envisaged as the most sensitive electrochemical technique for this kind of measurements. This work aims to provide an overview of the latest trends in the detection of contaminants by the simultaneous use of electrospun fibers and anodic stripping voltammetry. Indeed, a clear and comprehensive vision of the current status of this research may drive future improvements and new challenges.

## 1. Introduction

Electrospinning (ES) is a versatile technique applied to produce nano- and microfibers. It makes use of an electrical force to extrude conductive polymeric solutions in fibers with nanoscale diameters. With the help of a suitable polymeric vector, it is possible to produce one-dimensional (1D) nanostructures of organic, inorganic, and hybrid composite materials [1], with different morphologies and architectures, such as dense, hollow, core–shell, and fiber assemblies [2].

The fields of application of electrospun nanostructures are numerous and wide, considering the high versatility of this kind of material. Manifold examples can be found in the environmental sector, such as in water treatment from contaminants [3], in the catalysis field, as improved catalysts [4], in biomedical applications, as medicament carriers [5], in sensor devices, as high reactive sensing materials [6,7], and in the sustainable exploitation of energy, as innovative adsorbents [8,9,10,11]. Moreover, the ES technique has been intensively employed for the design and fabrication of structured nanofibrous materials for energy conversion and storage devices, as well as dye-sensitized solar cells [12], fuel cells [13], lithium/sodium-ion batteries [14], supercapacitors [15], and electronic applications in general [16].

Numerous electrospun materials have been realized and tested for heavy metal ions (HMIs) detection, exploiting very different approaches. For example, nanofibrous membranes immobilized in a chemosensor/receptor system were applied for optical, electro, and mass detections of heavy metals [17], and in other cases, the spectral changes, measured by an optic spectrometer, gave information on the detection process over colorimetric fibrous membranes [18]. Besides, electrospun fibers were also used in HMIs determination by conventional electrochemical measurements in aqueous media, such as cyclic voltammetry [19,20], and solid samples, such as electrokinetic remediation [21]. Indeed, electrospun materials have been successfully used for the detection of HMIs in different environments, such as wastewaters [22], rivers, and soils [21,23], on account of not appropriate wastes disposals and, in general, other causes linked to human activities. As heavy metals are in most cases non-biodegradable, they accumulate in foods derived from land animals, fishes, and vegetables. In turn, they are uptaken by humans, causing serious diseases. In this light, it is of vital importance to detect such species at very low traces and their neutralization [24].

Electrospun nanofibers possess many favorable characteristics in this sense, such as high surface-to-volume ratio, porosity, tunability of nanofibers properties (chemical composition, morphology, and dimensions), and they can be easily functionalized to optimize material performances [25].

Several techniques, generally considered as standard methods, have been utilized to detect traces of HMIs on well-known large-scale instruments, such as atomic absorption spectroscopy, inductive coupled plasma-mass spectroscopy, ion chromatography, x-ray fluorescence spectroscopy, and others [26,27,28]. These techniques, however, suffer from many disadvantages, i.e., long time analyses, expensiveness, complicated instrumentation, need of highly specialized personnel, and inability to carry out the on-site measurement. On the other hand, electrochemical techniques have been widely used on account of several advantages over the above-cited ones, such as high sensing performances, very low limits of detection (LOD), simpleness, fastness, and cheapness, together with the ability to simultaneously detect different substances [29,30]. Particularly, among these electrochemical methods, stripping voltammetry is a good choice for HMIs detection because of its simplicity, easy instrumentation, high sensitivity, low LOD, short-time measurement, and, last but not least, portability [31].

In this mini-review, we summarized the most recent advancements in the development of fibers, fabricated by ES, for the detection of HMIs by the anodic stripping voltammetry (ASV). Indeed, the coupling of electrospun nanofibers, characterized by the above-described advantages with those of the detection ability of the selected electrochemical method, is at the present, and maybe also in the next future, the winning solution for the HMIs sensing. Therefore, a brief and comprehensive overview of the current status of this research, not yet reported to the best of our knowledge, could provide new strategies and potential opportunities for the HMIs sensing, aiming at avoiding serious health issues for human beings.

Is out of the scope of the present work to review the countless papers dealing with the HMIs detection by the traditional techniques cited above, and materials and components other than nanofibers fabricated by ES. This topic has been recently faced in, where, however, other detection methodologies, besides the selected electrochemical one, were reviewed [16,17,32,33,34].

In the following, some fundamental concepts on the ES design and heavy metal toxicity were first introduced. Then, ASV principles and main methodologies, namely linear sweep, differential pulse, and square wave were summarized. Finally, the most recent findings on the use of electrospun fibers for the detection of contaminants employing ASV were reviewed.

## 2. Electrospinning Design

**Figure 1:**
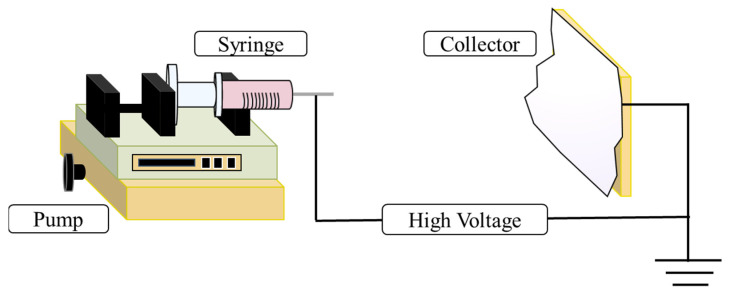
Schematic representation of the electrospinning set-up.

ES is considered a very simple, scalable, and inexpensive technique able to produce nanofibrous structures. It is based on the application of an electric field to a polymer carrier solution, conveniently introduced into a glass syringe and pushed through the syringe needle towards a grounded collector while controlling its flow rate, as depicted in Figure 1. The increment of the intensity of the electric field causes the drop of polymer to elongate and form a conical shape, the so-called Taylor cone. When the applied electric field reaches a critical value, the repulsive electric forces overcome the surface tension of the drop, the polymer jet is ironed within the high electric field and due to the rapid evaporation of the solvent is finally deposited on the collector in the form of nanofibers.

Accurate control of working parameters is essential to tailor nanofibers’ characteristics, in terms of diameters and morphologies [35]. Those parameters can be mainly divided into three areas covering solution, process, and ambient factors. Polymer solution parameters have the most significant influence on the ES process and include molecular weight, concentration, viscosity, conductivity, and surface tension of the solution. Processing parameters are indeed considered to have a major influence on fibers formation and take into account the applied voltage, flow rate, diameter of the needle, and tip-collector distance. Ambient parameters, such as temperature and humidity, are known to determine a great influence on the entire process. A summarized description of working parameters is reported in Table 1. Although the precise control of those parameters allows properties tunability and can therefore result in very different kinds of nanofibers, the well-known outstanding features are common. High porosity, small pore size, remarkable high surface area-to-volume ratio, and low density are surely among the most important.

In the ES process, a wide choice of natural, synthetic, and hybrid polymers is possible. Indeed, with the help of a suitable polymer vector, nanofibers of organic, inorganic, and hybrid composite materials with desired physical, chemical, and mechanical properties are produced [8,36,37]. The functionalization with suitable additives or focused chemical/thermal treatments can further convey superior and specific features. Consequently, the field of applications of electrospun nanofibers is large and varied and it should be also highlighted that the ES process, aside from its ease and cheapness, is overall flexible for large-scale production.

## 3. Contamination and Toxicity of Heavy Metals

**Figure 2:**
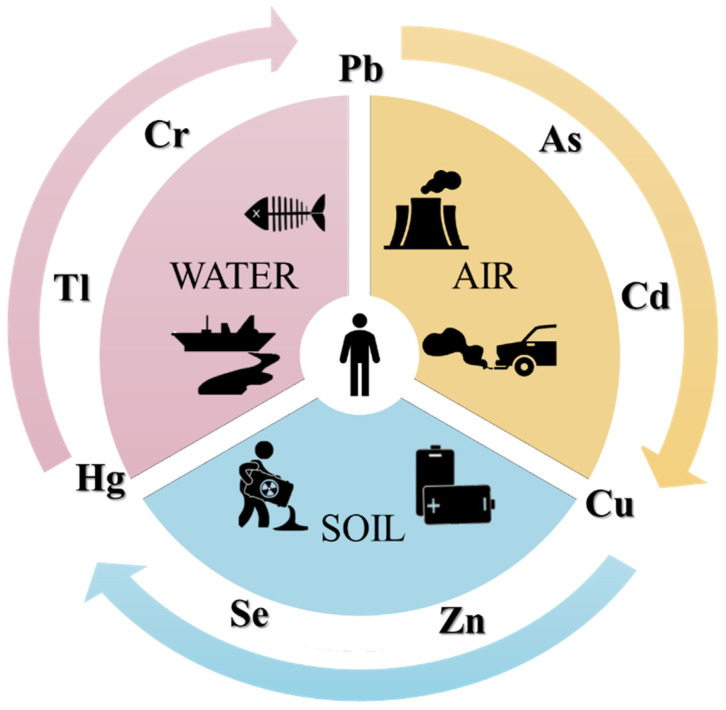
Schematic representation of heavy metals contamination in water, air, and soil environment.

The term heavy metal is generally ascribed to any metallic element based on featuring parameters such as the atomic weight/number, density, chemical behavior, and toxicity. Even if heavy metals are natural components of the Earth’s crust [38,39,40], they can also be the result of numerous anthropogenic activities such as mine tailings, disposal of high metal wastes, leaded gasoline, animal manures, land application of fertilizers, sewage sludge, pesticides, wastewater irrigation, coal combustion residues, and spillage of petrochemicals, causing soil, water, and air contamination [41], Figure 2. Typical heavy metals are arsenic (As), cadmium (Cd), chromium (Cr), lead (Pb), mercury (Hg), thallium (Tl), copper (Cu), selenium (Se), and zinc (Zn). The major problems that affect these metals are mainly related to the impossibility to be degraded or destroyed.

As a result, the increasing consumption in technological and industrial sectors has led to a wide distribution of heavy metals in the environment promoting their bioaccumulation. As trace elements, some heavy metals, i.e., zinc, selenium, copper, just to cite a few, are necessary for human metabolism [42], but they can cause poisoning at high concentrations. Indeed, when long time exposure to these substances is considered, many human diseases can arise and ascribe to them [43,44,45,46]. Considering that heavy metals pollution affects all environmental compartments (soil, water, and air), there are various routes through which they present risks to humans, animals, plants, and ecosystems as a whole. Such ways include alteration of pH soil, absorption by plants, consumption of contaminated water, and direct ingestion through the food chain [47]. Therefore, the toxicity of heavy metals depends on several parameters such as the route, the dose, the time of exposure, but it also depends on specific individuals (age, gender, and health state). In Table 2 the main features of heavy metals are summarized.

With these premises, the detection and the quantitative determination of HMIs in environmental compartments are of paramount importance. Heavy metals have been included as the priority substances to be monitored, especially in water, by several international organization like the World Health Organization (WHO), the US Environmental Protection Agency (EPA), Centre for Disease Control (CDC), and European Union (EU) [48,49,50,51].

## 4. Electrochemical Techniques for the Detection of Heavy Metal Ions

**Figure 3:**
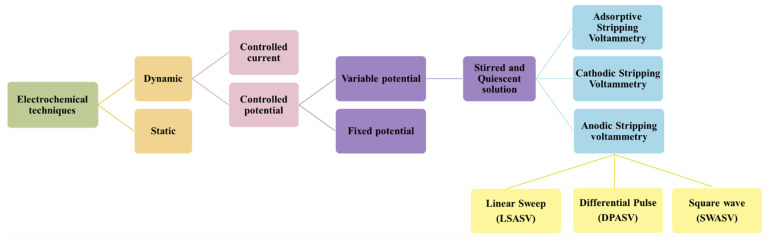
Electrochemical techniques.

The electrochemical techniques available for a different kind of analysis are numerous, as schematically depicted in Figure 3. The differences among them are mainly due to the LOD that can be achieved.

In HMIs detection, the need to have high sensitivity restricts the choice of the possible methods. In particular, stripping voltammetry is usually preferred.

Stripping voltammetry is a very sensitive analysis, highly indicated for the detection of trace metals in the ppb range. It is generally classified in anodic stripping voltammetry, cathodic stripping voltammetry, and adsorptive stripping voltammetry. Despite this, the anodic type is the most widely used.

ASV is regarded as a powerful electroanalytical technique and it is considered versatile and low cost [52]. Its main characteristic resides in the preconcentration of the analyte at the electrode followed by a potential sweep (voltammetry methods) to dissolve preconcentrated species of interest, making their quantification possible even at a low detection limit.

More than 20 elements, even simultaneously, can be detected with this method, reduced to the metallic state, and reoxidized. It consists of the formation of an amalgam due to the deposition of metal ions into a mercury-based electrode by the application of a sufficient potential to initiate reduction. In this configuration, while the applied potential is held constant the solution is stirred or otherwise, the electrode rotated for an established time. Finally, by scanning the applied potential in the opposite direction to the initial one, the amalgamated metals are stripped out of the mercury, that is to say oxidized. The peak currents obtained are proportional to the concentration of each metal in the solution and representative of the metal itself. The solubility of the analyte in mercury may influence the sensitivity. It is usually higher for metals that have higher solubilities, while in case of poor solubilities responses at the trace concentrations are not adequate. Mercury-free solid electrodes can also be used when it is necessary to detect elements that do not form amalgam or do not oxidize in combination with mercury.

Despite that this method is characterized by high sensitivity, there are even more possible actions to increase it. Indeed, according to the potential sweep, thus the waveforms produced, it is possible to identify linear sweep anodic stripping voltammetry (LSASV), differential pulse anodic stripping voltammetry (DPSAV), and square wave anodic stripping voltammetry (SWASV).

### 4.1. Linear Sweep Anodic Stripping Voltammetry

**Figure 4:**
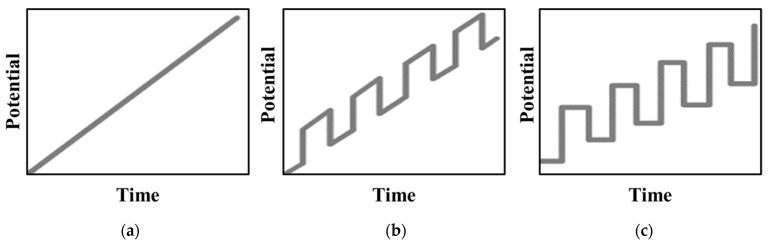
Profile of the potential versus time in (**a**) linear sweep, (**b**) differential pulse, and (**c**) square wave anodic stripping voltammetry (adapted from [62]).

In the LSASV, the voltage profile involves a linear scan in time, as depicted in Figure 4a. The current signal, corresponding to the potential at which the species begins to be oxidized or reduced, produces well-defined and selective stripping peaks, due to a lower interference among species. A wide range of metal ion concentrations can be detected with this method. In many cases, LSASV has been envisaged as a good alternative to another long and expensive method for lead detection [53,54] and for Zn, Pb, Cu, and Hg in trace quantity both alone and mixed all together [55]. The determination of Cr(VI) with the lowest limit of detection was instead reported by Aliyu et al. [56].

### 4.2. Differential Pulse Anodic Stripping Voltammetry

The improvement of detection limits is possible in ASV when using potential waveforms other than the linear sweep. DPASV uses a staircase potential waveform superimposed on the linear waveform of linear sweep voltammetry, as shown in Figure 4b.

**Figure 5:**
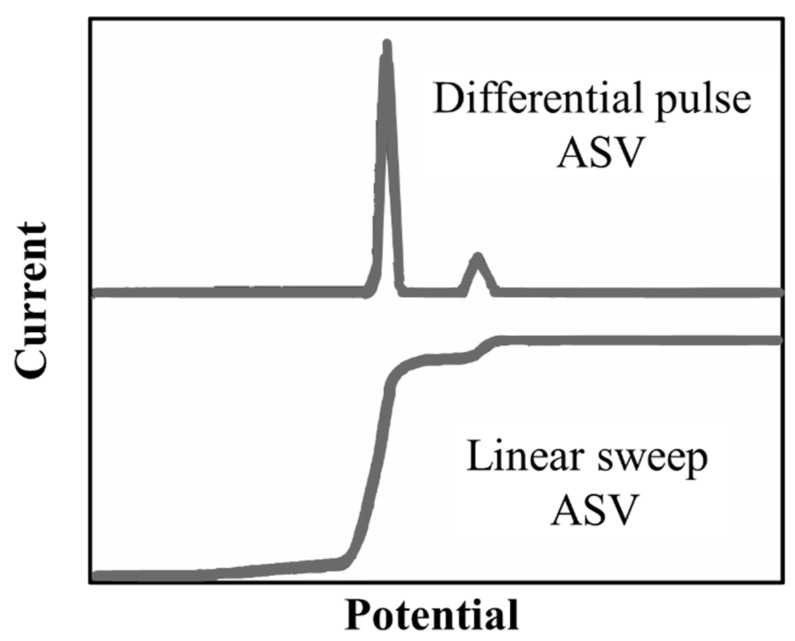
Profile of the current versus the potential for the linear sweep and differential pulse voltammograms in the presence of two contaminants.

The differential pulse method is much more sensitive compared to the linear one. It provides a sharp peak for each species being analyzed and, as a consequence, the measure of the height or the area of these peaks results to be more precise, as its relation to the concentration is improved. Indeed, a small peak in the differential pulse mode, due to low concentrations, is much simpler to measure than only a small change in the height of the current, as depicted in Figure 5.

Moreover, it is much easier to distinguish two species with similar potential, considering that it only requires a difference of about 0.05 V contrary to LSASV where a difference of about 0.2 V is necessary.

Multiple heavy metals, in very low concentrations, have been successfully detected by this method, as extensively reported in the literature, where the advantages of the technique were clearly exploited [57,58,59].

### 4.3. Square Wave Anodic Stripping Voltammetry

If the potential waveform is a symmetrical square wave superimposed on a staircase potential, then SWASV is considered, Figure 4c. It is an extension of the square wave voltammetry, since it works the same, except for the preconcentration step, typical of all the stripping analysis. During each square wave cycle, the current is sampled twice, respectively at the end of the forward and reverse pulse. The difference in current between the two points is plotted versus the applied potential.

Being currents very high, LOD can achieve sensitivity in the parts-per-trillion range, especially when more than one metal ion in the sample is present.

Indeed, due to the high sensitivity and selectivity, it is generally preferred in the detection of heavy metals [60,61].

## 5. Recent Nanofibers for HMIs Sensors

In the last years, novel electrospun nanofibrous membranes acting as electrochemical sensors for HMIs detection have been manufactured. Very different types of electrospun nanofibrous membranes were considered, such as pure or doped, hybrid, and composite, demonstrated themselves as highly recommended materials in this sensing field, especially when their performances were compared with the same material synthesized with a different method.

The removal of heavy metals compounds from contaminated air, soil, and water using electrospun fibers is mainly due to the interactions between the active sites on the surface of fibers and HMIs, originated by electrostatic interactions and both physical and chemical affinity. Briefly, the functional sites of which fibers can be endowed, through the different functionalization or doped methods, promote the activation of anchor points for HMIs. Moreover, the high surface area to volume ratio of fibers conveys a broader dispersion of active sites, that along with the porous structure of fibrous membranes, assess the heavy metal removal capability of the electrospun [63].

Therefore, it has experimented that the integration of nanomaterials as electrodes can improve the sensitivity and reproducibility of electrochemical methods in the detection of HMIs [64].

When ASV is used, the preconcentration of the analyte at the electrode surface, by both static and dynamic adsorption, is first involved and then followed by a potential sweep that, through the oxidation/reduction process, determines the detection mechanism.

**Figure 6:**
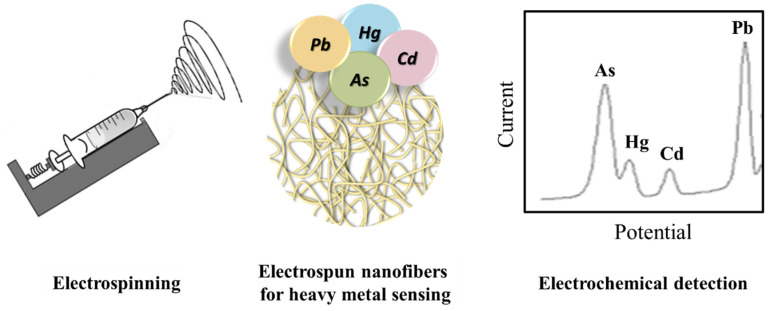
Representative sketch of the analyzed combined system.

In this way, considering the advantageous combination of electrochemical techniques and nanofibrous materials (Figure 6), smaller electrochemical signal detectors, such as smart and field-portable devices, can be easily developed to replace classical bulky electrochemical workstations [31].

Limited examples of this combined system can be found in the literature. In particular, when polymeric nanofibers are considered, polyaniline conductive polymer was evidenced among the most used sources. Indeed, it played a dual role in both increasing the surface area, due to the monodimensional structure, and assuring at the same time a high conductivity due to the well-known electronic properties. Promphet et al. [65] reported electrospun nanoporous fibers fabricated by mixing graphene, polyaniline, and polystyrene and deposited onto carbon electrodes. The system was tested under SWASV for the simultaneous determination of Pb^2+^ and Cd^2+^ ions in the presence of Bi^3+^ ions. The observed increase in the electrochemical sensitivity of modified electrodes (observed in comparison to unmodified ones) was ascribed to an increase of the specific electrode surface area because of the presence of nanofibers. As usual, the ES process parameters (type of solvent, tip to collector distance, and electric field between syringe and collector) played a pivotal role in determining the fibers morphology and electrochemical sensitivity. The electrode system showed high reproducibility and was successfully applied in real river water samples, demonstrating to be a useful tool for the environmental monitoring of these ions with a detection limit for Pb^2+^ and Cd^2+^ down to 3.3 and 4.43 μg/L, respectively. Huang et al. [66] used one-dimensional phytic acid doped polyaniline nanofibers as a modifier for glassy carbon electrodes for simultaneous detection of Cd^2+^ and Pb^2+^ using DPASV. The reported detection limits were 0.02 and 0.05 µg/L for Cd^2+^ and Pb^2+^, respectively. The fabrication and evaluation of a glassy carbon electrode modified with self-doped polyaniline nanofibers/mesoporous carbon nitride and bismuth for simultaneous determination of trace Cd^2+^ and Pb^2+^ by SWASV were reported by Zhang et al. [67]. Under the optimum conditions, the fabricated electrode exhibited LOD of 0.7 nM for Cd^2+^ and 0.2 nM for Pb^2+^.

Both individual and simultaneous detection of Pb^2+^ and Cd^2+^ were afterward investigated on polyaniline nanotubes/electrospun polystyrene surface [68]. Morphological and electrochemical properties of the PANI nanotubes were demonstrated to depend on the size of the polystyrene fibers. Good sensitivity and reproducibility were demonstrated by SWASV. The LOD was found to be 9.65 × 10^−11^ mol/L and 2.67 × 10^−10^ mol/L for the individual detection of Pb^2+^and Cd^2+^ ions, respectively. Simultaneous detection of Zn^2+^, Cd^2+^, and Pb^2+^ using graphene–polyaniline nanocomposite electrode was investigated by SWASV in the presence of Bi^3+^. Metal ion concentration with detection limits of 1.0 μg/L for Zn^2+^ and 0.1 μg/L for both Cd^2+^ and Pb^2+^. The modified electrode allowed selective determination of the target metals in the presence of common metal interferences including Mn^2+^, Cu^2+^, Fe^3+^, Fe^2+^, Co^3+^, and Ni^2+^ [69]. Field monitoring of HMIs pollution for the detection of Hg^2+^ was demonstrated by Naourei et al. [70]. A novel conductive nanofibrillar structure made of a copolymer, poly(aniline-co-*o*-aminophenol) decorated with gold nanoparticles allowed mercury ions quantification by ASV with a very LOD of 0.23 nM. The sensor resulted efficient in Hg^2+^ detection in river water and in fish samples.

Along with electrospun polyaniline, another commonly reported system was based on carbon nanofibers, considered a system with remarkable surface area. Li 2010 et al. [71] constructed a bismuth-film modified graphite nanofibers–Nafion glassy carbon electrode for the simultaneous determination of trace Cd^2+^ and Pb^2+^. The electrochemical properties of the modified electrode, studied under optimal conditions, showed the LOD equal to 0.09 μg/L for Cd^2+^ and 0.02 μg/L for Pb^2+^ with a 10 min preconcentration by DPASV. High reproducibility and selectivity, in both real, such as river water, and human blood samples were demonstrated. The same metals, generally reported as representative metals, were also used by Zhao et al. [72] to test through the same electrochemical technique the performance of a carbon-based fibers/Nafion modified electrode, resulting in a LOD equal to 0.9 × 10^−9^ M and 1.5 × 10^−9^ M, for Pb^2+^ and Cd^2+^, respectively. A Nafion-modified glassy carbon electrode, combining bis(indolyl)methane with the unique properties of mesoporous carbon nanofiber, was fabricated for anodic stripping analysis of Hg^2+^ with a satisfactory result in HMIs coexistence (Hg^2+^, Cd^2+^, Pb^2+^, and Cu^2+^). This electrochemical sensor significantly improved selectivity and sensitivity towards Hg^2+^ determination with a detection limit of 0.3 nM [73]. Glutathione peptide was successfully immobilized onto a nanomaterial substrate electrode based on the carbon nanofiber. The repeatability, reproducibility, and analytical performance were compared to a classical screen-printed carbon electrode modified with glutathione. It was demonstrated that the enhanced surface area attributed to carbon nanofibers substrate gave higher results to Pb^2+^ and Cd^2+^ determination [74]. A facile, green, highly sensitive, and simultaneous method for the detection of trace HMIs was demonstrated by Zhang et al. [75]. Well-dispersed gold nanoparticles, grown on carbon nanofibers synthesized via the ES technique and exhibited a high surface-specific area and high porosity. When used as the working electrode for the simultaneous determination of HMIs such as Cd^2+^, Pb^2+^, and Cu^2+^ through the SWASV method, those properties resulted in being beneficial for the penetration of heavy metal ion solutions and increased the surface contact area of the electrode. Simultaneous detection of Cd^2+^, Pb^2+^, and Cu^2+^ with a low concentration of 0.1 mM, was attributed to the exposed well-dispersed gold nanoparticles and the high electron transport capability of carbon nanofibers. Vertically aligned carbon nanofibers were evaluated for the detection of Pb^2+^ by ASV by Robinson et al. [76]. The achieved detection limit reported was 1.73 nM. A nanocomposite of polypyrrole and carbon nanofibers modified carbon paste electrode was reported for the determination of traces of lead ions in real samples of water. The SWASV was used to investigate the analytical performances of the designed electrode. Under the optimum experimental conditions, good linearity between the stripping peak currents and the concentration of Pb^2+^ was obtained with a detection limit estimated around 0.05 μg/L [77]. Another research provides a convenient and efficient way to construct new sensors for simultaneous trace detection of HMIs. Bimetallic platinum-gold alloy nanoparticles were constructed with electrospun carbon nanofibers for simultaneous detection of trace Cd^2+^, Pb^2+^, and Cu^2+^ by SWASV with a sensitivity of 0.10 mM. The excellent sensitivity and low detection limit for HMIs detection were ascribed to the high conductivity of CNFs, fast response of platinum-gold alloy nanoparticles, and high specific surface area of the hybrid structure [78]. Porous carbon nanofibers co-doped with nitrogen and sulfur were used to modify a glassy carbon electrode. Compared to a bare glassy carbon electrode, the one modified shows improved sensitivity for Cd^2+^ in DPASV [79]. A novel elaborated electrochemical sensor based on a nanocomposite of ionic liquid 1-ethyl-3-methylimidazolium bis(trifluoromethylsulfonyl) imide, carbon nanofibers, and bismuth particles modified carbon paste was studied. The sensor exhibited excellent electroanalytical performances for the determination of Pb^2+^ and Cd^2+^ with LOD for Pb^2+^ and Cd^2+^ equal to 0.12 and 0.25 μg/L, respectively. A wide linear response range and low LOD with high sensitivity, selectivity, and reproducibility were obtained [80]. A three-dimensional carbon nanofiber network formed by the ES of a mixed solution of montmorillonite and polyacrylonitrile showed high stability, selectivity, and a low detection limit (0.4 μg/L) for Cu^2+^ as measured using DPASV [81]. A trace amount of As^3+^ detection was instead reported by using a sensing platform made by polyaniline nanosheet array on electrospun iron-containing carbon nanofibers substrate followed by self-deposition of gold nanoparticles. High sensitivity with a detection limit of 0.5 ppb was registered by SWASV [82].

Few other works reported on other kinds of pure, hybrid, and composite nanofibers for multiple HMIs detection. Reduced graphene oxide/polyvinyl butyral nanofibers on glassy carbon electrodes were used for fabricating an electrochemical sensor for Cu^2+^ detection by DPASV. The as-fabricated sensor showed good analytical performance with a low detection limit of 4.1 nM [83]. Zinc oxide nanofibers with high surface area, fabricated by ES, have demonstrated optimum sensitivity for Cd^2+^ and the simultaneous analysis with Cu^2+^, Pb^2+^, Cu^2+^, and Hg^2+^ is feasible in water samples. The strong affinity between zinc oxide and Cd^2+^ was exploited by SWASV and the reported LOD was equal to 1.8·10^−9^mol/L [84]. A zinc oxide nanofibers/L-cysteine nanocomposite was used as electrodes for the electrochemical sensing of Pb^2+^ ions. The high porosity of nanofibers combined with Lewis acid-base interaction of L-cysteine made it a great material for Pb^2+^ detection in a real sample. SWASV displayed excellent sensitivity, selectivity, and stability with a LOD of 0.397 μg/L [85]. To test Cu^2+^ ions contaminations in real environmental samples, an amino-rich organosilica-nanofiber-modified gold electrode was obtained upon ES. The 3-dimensional porous fiber promoted Cu^2+^ ions accumulation inside the structure. By using SWASV, a very sensitive response to Cu^2+^ ions was obtained, with a wide linear range, high stability, and low detection limit down to 2.6 pM [86].

Finally, it is noteworthy to highlight that, as inferred by the analyzed literature, the pivotal role in the sensing mechanism of HMIs by ASV was strictly dependent on the material features. Despite a very broad diameters distribution, ranging from 40–60 nm [66] to 2.44 μm [65] was evidenced for the reported cases, on account of different electrospun fibers (such as pure, hybrid, doped, and functionalized), the high surface area to volume ratio of fibers and membranes porosity were always ensured for each system.

## 6. Concluding Remarks

The application of ASV for the determination of HMIs using electrospun nanofibers is undoubtedly a well-assessed field of research. Heavy metals pollution is a pressing issue since it involves water, air, and soil contamination and consequently, it also results harmful to humankind. When high selectivity and very low traces of contaminants are studied, the combination materials/methods showed a great impact. ASV was evaluated in its main potential configurations, linear sweep, differential pulse, and square wave, whereas manifold electrospun structures, pure, doped, composite, and hybrid were considered. Indeed, the outstanding properties of electrospun fibers, in terms of their tunability by proper doping and functionalization, but also their high surface area to volume ratio and porosity, were opportunely revised for the specific application in the ASV of heavy metals.

However, the design and development of focused strategies for the effective detection of HMIs is still a big issue and needs further improvements. Through this contribution, an overview of the current trends in the detection of HMIs by ASV and electrospun nanofibers was provided. The review aims to drive future challenges in the enhancements of fibrous materials for both the detection and remediation of heavy metals in a sustainable perspective of a circular economy.

## Figures and Tables

**Table 1:** Main electrospinning working parameters.

Electrospinning Working Parameters		
Solution	Process	Ambient
Molecular weight Concentration Viscosity Conductivity Surface tension	Applied voltage Flow rate Diameter of the needle Tip-collector distance	Temperature Humidity

**Table 2:** Sources of heavy metals and toxicity on human health.

Heavy Metals	Sources	Toxic Impact on Human Health	Permissible Limits [49,50]
**As**	drinking-water and food; industrial processes; tobacco	pigmentation changes, skin lesions, hyperkeratosis	10 μg/L
**Cd**	corrosion of galvanized pipes, erosion of natural deposits, discharge from metal refineries, runoff from waste batteries and paints	renal tubular dysfunction, osteoporosis, acute pneumonitis, cancer	3 μg/L
**Cr**	coal and oil combustion; electroplating; leather tanning; industrial processes, tobacco	contact dermatitis, hemolysis, renal diseases, allergic reaction, cancer	50 μg/L
**Pb**	drinking-water and food; lead-containing pipes; children’s toys; cosmetics	microcytic anemia; nephropathy; immune system diseases; reproductive system diseases; developmental system disease	10 μg/L
**Hg**	accidents, environmental pollution, dental care, preventive medical practices, industrial and agricultural operations, fish consumption	calcium homeostasis; neurological diseases, corrosive to skin, corrosive to eyes, cancer, corrosive to the gastrointestinal tract	6 μg/L
**Tl**	drinking-water and food; air-borne contamination (fly ash)	gaseous emission of cement factories, coal-burning power plants, metal sewers	0.5 µg/L
**Se**	natural deposits releases from copper smelting	endocrine function, hepatotoxicity, and gastrointestinal disturbances	40 μg/L
**Cu**	industrial discharges, copper salts, plumbing material	brain and kidney damage, chronic anemia, stomach and intestine irritation	2 mg/L
**Zn**	discharges of smelter slags and wastes, mine tailings, coal and bottom fly ash, fertilizers	dizziness, fatigue	5 mg/L

## Data Availability

Not applicable.

## References

[1] Toriello M., Afsari M., Shon H.K., Tijing L.D. (2020). Progress on the Fabrication and Application of Electrospun Nanofiber Composites. Membranes.

[2] Song J., Yan W., Cao H., Song Q., Ding H., Lv Z., Zhang Y., Sun Z. (2019). Material flow analysis on critical raw materials of lithium-ion batteries in China. J. Clean. Prod..

[3] Zander N.E., Gillan M., Sweetser D. (2016). Recycled PET Nanofibers for Water Filtration Applications. Materials.

[4] Malara A., Paone E., Bonaccorsi L., Mauriello F., Macario A., Frontera P. (2019). Pd/Fe_3_O_4_ Nanofibers for the Catalytic Conversion of Lignin-Derived Benzyl Phenyl Ether under Transfer Hydrogenolysis Conditions. Catalysts.

[5] Ferrández-Rives M., Beltrán-Osuna Á.A., Gómez-Tejedor J.A., Ribelles J.L.G. (2017). Electrospun PVA/Bentonite Nanocomposites Mats for Drug Delivery. Materials.

[6] Zhang Q., Wang X., Fu J., Liu R., He H., Ma J., Yu M., Ramakrishna S., Long Y. (2018). Electrospinning of Ultrafine Conducting Polymer Composite Nanofibers with Diameter Less than 70 nm as High Sensitive Gas Sensor. Materials.

[7] Frontera P., Trocino S., Donato A., Antonucci P.L., Faro M.L., Squadrito G., Neri G. (2014). Oxygen-sensing properties of electrospun CNTs/PVAc/TiO_2_ composites. Electron. Mater. Lett..

[8] Malara A., Frontera P., Bonaccorsi L., Antonucci P.L. (2018). Hybrid Zeolite SAPO-34 Fibres Made by Electrospinning. Materials.

[9] Frontera P., Kumita M., Malara A., Nishizawa J., Bonaccorsi L. (2019). Manufacturing and Assessment of Electrospun PVP/TEOS Microfibres for Adsorptive Heat Transformers. Coatings.

[10] Freni A., Calabrese L., Malara A., Frontera P., Bonaccorsi L. (2019). Silica gel microfibres by electrospinning for adsorption chillers. Energy.

[11] Bonaccorsi L., Fotia A., Malara A., Frontera P. (2020). Advanced Adsorbent Materials for Waste Energy Recovery. Energies.

[12] López-Covarrubias J.G., Soto-Muñoz L., Iglesias A.L., Villarreal-Gómez L.J. (2019). Electrospun Nanofibers Applied to Dye Solar Sensitive Cells: A Review. Materials.

[13] Aruna S., Balaji L., Kumar S.S., Prakash B.S. (2017). Electrospinning in solid oxide fuel cells—A review. Renew Sustain. Energy Rev..

[14] Malara A., Pantò F., Santangelo S., Antonucci P.L., Fiore M., Longoni G., Ruffo R., Frontera P. (2021). Comparative life cycle assessment of Fe2O3-based fibers as anode materials for sodium-ion batteries. Environ. Dev. Sustain..

[15] Bhattacharya S., Roy I., Tice A., Chapman C., Udangawa R.N., Chakrapani V., Plawsky J.L., Linhardt R.J. (2020). High-Conductivity and High-Capacitance Electrospun Fibers for Supercapacitor Applications. ACS Appl. Mater. Interfaces.

[16] Luzio A., Canesi E.V., Bertarelli C., Caironi M. (2014). Electrospun Polymer Fibers for Electronic Applications. Materials.

[17] Zhang N., Qiao R., Su J., Yan J., Xie Z., Qiao Y., Wang X., Zhong J. (2017). Recent Advances of Electrospun Nanofibrous Membranes in the Development of Chemosensors for Heavy Metal Detection. Small.

[18] Balusamy B., Senthamizhan A., Uyar T. (2020). Functionalized Electrospun Nanofibers as a Versatile Platform for Colorimetric Detection of Heavy Metal Ions in Water: A Review. Materials.

[19] Musameh M.M., Klink D., Choi J., Truong Y.B., Kyratzis I.L. (2012). Electrochemical Detection of Metals Using Thick Nanofibrous Nafion Web Modified Electrodes. Electroanalysis.

[20] Teodoro K.B., Migliorini F.L., Facure M.H., Correa D.S. (2019). Conductive electrospun nanofibers containing cellulose nanowhiskers and reduced graphene oxide for the electrochemical detection of mercury(II). Carbohydr. Polym..

[21] Wang J., Hou L., Yao Z., Jiang Y., Xi B., Ni S., Zhang L. (2021). Aminated electrospun nanofiber membrane as permeable reactive barrier material for effective in-situ Cr(VI) contaminated soil remediation. Chem. Eng. J..

[22] Karkra R., Kumar P., Bansod B.K.S., Bagchi S., Sharma P., Krishna C.R. (2017). Classification of heavy metal ions present in multi-frequency multi-electrode potable water data using evolutionary algorithm. Appl. Water Sci..

[23] Di Natale C., Macagnano A., Davide F., D’Amico A., Legin A., Vlasov Y., Rudnitskaya A., Selezenev B. (1997). Multicomponent analysis on polluted waters by means of an electronic tongue. Sens. Actuators B Chem..

[24] Malara A., Paone E., Frontera P., Bonaccorsi L., Panzera G., Mauriello F. (2018). Sustainable Exploitation of Coffee Silverskin in Water Remediation. Sustainability.

[25] Fotia A., Malara A., Paone E., Bonaccorsi L., Frontera P., Serrano G., Caneschi A. (2021). Self Standing Mats of Blended Polyaniline Produced by Electrospinning. Nanomaterials.

[26] Ipeaiyeda A.R., Ayoade A.R. (2017). Flame atomic absorption spectrometric determination of heavy metals in aqueous solution and surface water preceded by co-precipitation procedure with copper(II) 8-hydroxyquinoline. Appl. Water Sci..

[27] Ammann A.A. (2001). Speciation of heavy metals in environmental water by ion chromatography coupled to ICP–MS. Anal. Bioanal. Chem..

[28] McComb J.Q., Rogers C., Han F.X., Tchounwou P.B. (2014). Rapid Screening of Heavy Metals and Trace Elements in Environmental Samples Using Portable X-Ray Fluorescence Spectrometer, A Comparative Study. Water Air Soil Pollut..

[29] Frontera P., Malara A., Stelitano S., Leonardi S.G., Bonavita A., Fazio E., Antonucci P., Neri G., Neri F., Santangelo S. (2016). Characterisation and H_2_O_2_ sensing properties of TiO_2_ -CNTs/Pt electro-catalysts. Mater. Chem. Phys..

[30] Malara A., Leonardi S., Bonavita A., Fazio E., Stelitano S., Neri G., Neri F., Santangelo S. (2016). Origin of the different behavior of some platinum decorated nanocarbons towards the electrochemical oxidation of hydrogen peroxide. Mater. Chem. Phys..

[31] Li Y., Chen Y., Yu H., Tian L., Wang Z. (2018). Portable and smart devices for monitoring heavy metal ions integrated with nanomaterials. TrAC Trends Anal. Chem..

[32] Buledi J.A., Amin S., Haider S.I., Bhanger M.I., Solangi A.R. (2020). A review on detection of heavy metals from aqueous media using nanomaterial-based sensors. Environ. Sci. Pollut. Res..

[33] Jin M., Yuan H., Liu B., Peng J., Xu L., Yang D.-Z. (2020). Review of the distribution and detection methods of heavy metals in the environment. Anal. Methods.

[34] Li M., Gou H., Al-Ogaidi I., Wu N. (2013). Nanostructured Sensors for Detection of Heavy Metals: A Review. ACS Sustain. Chem. Eng..

[35] Li Z., Wang C., Li Z., Wang C. (2013). Effects of Working Parameters on Electrospinning BT—One-Dimensional nanostructures: Electrospinning Technique and Unique Nanofibers.

[36] Frontera P., Busacca C., Trocino S., Antonucci P., Faro M.L., Falletta E., Della Pina C., Rossi M. (2013). Electrospinning of polyaniline: Effect of different raw sources. J. Nanosci. Nanotechnol..

[37] Frontera P., Malara A., Stelitano S., Fazio E., Neri F., Scarpino L., Antonucci P.L., Santangelo S. (2015). A new approach to the synthesis of titania nano-powders enriched with very high contents of carbon nanotubes by electro-spinning. Mater. Chem. Phys..

[38] Fergusson J.E. (1990). The Heavy Elements: Chemistry, Environmental Impact and Health Effects.

[39] Duffus J.H. (2002). “Heavy metals” a meaningless term? (IUPAC Technical Report). Pure Appl. Chem..

[40] Bradl H. (2005). Chapter 1 Sources and origins of heavy metals. Heavy Metals in the Environment: Origin, Interaction and Remediation.

[41] Mayer R. (1982). NW Lepp (Ed.): Effect of heavy metal pollution on plants. Vol. 1: Effects of trace metals on plant function. 352 S. Vol. 2: Metals in the environment. 257 S. Applied Science Publishers, London and New Jersey, 1981. £ 26.00 (Vol. 1) und £ 21.00 (Vol. 2). J. Plant. Nutr. Soil Sci..

[42] Chan S., Gerson B., Subramaniam S. (1998). The Role of Copper, Molybdenum, Selenium, and Zinc in Nutrition and Health. Clin. Lab. Med..

[43] Bandmann O., Weiss K.H., Kaler S.G. (2015). Wilson’s disease and other neurological copper disorders. Lancet Neurol..

[44] Prystupa A., Błażewicz A., Kiciński P., Sak J.J., Niedziałek J., Załuska W. (2016). Serum Concentrations of Selected Heavy Metals in Patients with Alcoholic Liver Cirrhosis from the Lublin Region in Eastern Poland. Int. J. Environ. Res. Public Health.

[45] Hadrup N., Ravn-Haren G. (2020). Acute human toxicity and mortality after selenium ingestion: A review. J. Trace Elem. Med. Biol..

[46] Kim H.S., Kim Y.J., Seo Y.R. (2015). An Overview of Carcinogenic Heavy Metal: Molecular Toxicity Mechanism and Prevention. J. Cancer Prev..

[47] Musilova J., Arvay J., Vollmannova A., Toth T., Tomas J. (2016). Environmental Contamination by Heavy Metals in Region with Previous Mining Activity. Bull. Environ. Contam. Toxicol..

[48] Tchounwou P.B., Yedjou C.G., Patlolla A.K., Sutton D.J. (2012). Heavy Metal Toxicity and the Environment. Galanin.

[49] Edition F. (2011). Guidelines for drinking-water quality. WHO Chron..

[50] Tiemann M. (2014). Safe Drinking Water Act (SDWA): A Summary of the Act and Its Major Requirements.

[51] Tappero J.W., Cassell C.H., Bunnell R.E., Angulo F.J., Craig A., Pesik N., Dahl B.A., Ijaz K., Jafari H., Martin R. (2017). US Centers for Disease Control and Prevention and Its Partners’ Contributions to Global Health Security. Emerg. Infect. Dis..

[52] Borrill A.J., Reily N.E., Macpherson J. (2019). V Addressing the practicalities of anodic stripping voltammetry for heavy metal detection: A tutorial review. Analyst.

[53] Mazerie I., Geneste F. (2020). Coupling of Anodic Stripping Voltammetry with Sampled-Current Voltammetry on an Electrode Array: Application to Lead Detection. Sensors.

[54] Mouhamed N., Cheikhou K., Elhadji G., Bagha D.M., Guèye M.-D.C., Tzedakis T. (2018). Determination of Lead in Water by Linear Sweep Anodic Stripping Voltammetry (LSASV) at Unmodified Carbon Paste Electrode: Optimization of Operating Parameters. Am. J. Anal. Chem..

[55] Khun N., Liu E. (2009). Linear sweep anodic stripping voltammetry of heavy metals from nitrogen doped tetrahedral amorphous carbon thin films. Electrochim. Acta.

[56] Tukur S.A., Yusof N.A., Hajian R. (2015). Linear sweep anodic stripping voltammetry: Determination of Chromium (VI) using synthesized gold nanoparticles modified screen-printed electrode. J. Chem. Sci..

[57] Thanh N.M., Van Hop N., Luyen N.D., Phong N.H., Thanh T., Toan T. (2019). Simultaneous Determination of Zn (II), Cd (II), Pb (II), and Cu (II) Using Differential Pulse Anodic Stripping Voltammetry at a Bismuth Film-Modified Electrode. Adv. Mater. Sci. Eng..

[58] Manisankar P., Vedhi C., Selvanathan G., Arumugam P. (2008). Differential pulse stripping voltammetric determination of heavy metals simultaneously using new polymer modified glassy carbon electrodes. Microchim. Acta.

[59] Da Silveira T., Araújo D., Marchini L., Moreti A., Olinda R. (2013). Detection of metals by differential pulse anodic stripping voltammetry (DPASV) in pollen collected from a fragment of the atlantic forest in Piracicaba/SP. Ecotoxicol. Environ. Contam..

[60] He X., Chen L., Xie X., Su Z., Qin C., Liu Y., Ma M., Yao S., Deng L., Xie Q. (2011). Square wave anodic stripping voltammetric determination of lead(II) using a glassy carbon electrode modified with a lead ionophore and multiwalled carbon nanotubes. Microchim. Acta.

[61] Farghaly O.A., Ghandour M. (2005). Square-wave stripping voltammetry for direct determination of eight heavy metals in soil and indoor-airborne particulate matter. Environ. Res..

[62] Segura B., Jiménez F.N., Giraldo L.R. (2016). Potentiostat prototype with applications in electrochemical processes. Entre Cienc. Ing..

[63] Huang Y., Miao Y.-E., Liu T. (2014). Electrospun fibrous membranes for efficient heavy metal removal. J. Appl. Polym. Sci..

[64] Liu Y., Deng Y., Dong H., Liu K., He N. (2017). Progress on sensors based on nanomaterials for rapid detection of heavy metal ions. Sci. China Ser. B Chem..

[65] Promphet N., Rattanarat P., Rangkupan R., Chailapakul O., Rodthongkum N. (2015). An electrochemical sensor based on graphene/polyaniline/polystyrene nanoporous fibers modified electrode for simultaneous determination of lead and cadmium. Sens. Actuators B Chem..

[66] Huang H., Zhu W., Gao X., Liu X., Ma H. (2016). Synthesis of a novel electrode material containing phytic acid-polyaniline nanofibers for simultaneous determination of cadmium and lead ions. Anal. Chim. Acta.

[67] Zhang C., Zhou Y., Tang L., Zeng G., Zhang J., Peng B., Xie X., Lai C., Long B., Zhu J. (2016). Determination of Cd^2+^ and Pb^2+^ Based on Mesoporous Carbon Nitride/Self-Doped Polyaniline Nanofibers and Square Wave Anodic Stripping Voltammetry. Nanomaterials.

[68] Zhu G., Ge Y., Dai Y., Shang X., Yang J., Liu J. (2018). Size-tunable polyaniline nanotube-modified electrode for simultaneous determination of Pb(II) and Cd(II). Electrochim. Acta.

[69] Ruecha N., Rodthongkum N., Cate D.M., Volckens J., Chailapakul O., Henry C.S. (2015). Sensitive electrochemical sensor using a graphene–polyaniline nanocomposite for simultaneous detection of Zn(II), Cd(II), and Pb(II). Anal. Chim. Acta.

[70] Narouei F.H., Livernois L., Andreescu D., Andreescu S. (2021). Highly sensitive mercury detection using electroactive gold-decorated polymer nanofibers. Sens. Actuators B Chem..

[71] Li D., Jia J., Wang J. (2010). Simultaneous determination of Cd(II) and Pb(II) by differential pulse anodic stripping voltammetry based on graphite nanofibers–Nafion composite modified bismuth film electrode. Talanta.

[72] Zhao D., Wang T., Han D., Rusinek C., Steckl A.J., Heineman W.R. (2015). Electrospun Carbon Nanofiber Modified Electrodes for Stripping Voltammetry. Anal. Chem..

[73] Liao Y., Li Q., Wang N., Shao S. (2015). Development of a new electrochemical sensor for determination of Hg(II) based on Bis(indolyl)methane/Mesoporous carbon nanofiber/Nafion/glassy carbon electrode. Sens. Actuators B Chem..

[74] Ràfols C.P., Serrano N., Díaz-Cruz J.M., Ariño C., Esteban M. (2016). Glutathione modified screen-printed carbon nanofiber electrode for the voltammetric determination of metal ions in natural samples. Talanta.

[75] Zhang B., Chen J., Zhu H., Yang T., Zou M., Zhang M., Du M. (2016). Facile and green fabrication of size-controlled AuNPs/CNFs hybrids for the highly sensitive simultaneous detection of heavy metal ions. Electrochim. Acta.

[76] Robinson J.E., Heineman W.R., Sagle L.B., Meyyappan M., Koehne J.E. (2016). Carbon nanofiber electrode array for the detection of lead. Electrochem. Commun..

[77] Oularbi L., Turmine M., El Rhazi M. (2017). Electrochemical determination of traces lead ions using a new nanocomposite of polypyrrole/carbon nanofibers. J. Solid State Electrochem..

[78] Zhang S., Zhu H., Ma P., Duan F., Dong W., Du M. (2017). A self-supported electrochemical sensor for simultaneous sensitive detection of trace heavy metal ions based on PtAu alloy/carbon nanofibers. Anal. Methods.

[79] Gao S., Liu J., Luo J., Mamat X., Sambasivam S., Li Y., Hu X., Wågberg T., Hu G. (2018). Selective voltammetric determination of Cd(II) by using N,S-codoped porous carbon nanofibers. Microchim. Acta.

[80] Oularbi L., Turmine M., Salih F.E., El Rhazi M. (2020). Ionic liquid/carbon nanofibers/bismuth particles novel hybrid nanocomposite for voltammetric sensing of heavy metals. J. Environ. Chem. Eng..

[81] Cheng H., Zhou Z., Qin D., Huang W., Feng J., Tang T., Hu G., Li L. (2020). Electrochemical Sensor Based on Electrospun Three-Dimensional Carbon Nanofibers to Determine Trace Levels of Cu(II). Sci. Adv. Mater..

[82] Tang Q., Zhu G., Ge Y., Yang J., Huang M., Liu J. (2020). AuNPs-polyaniline nanosheet array on carbon nanofiber for the determination of As(III). J. Electroanal. Chem..

[83] Ding R., Luo Z., Ma X., Fan X., Xue L., Lin X., Chen S. (2015). High Sensitive Sensor Fabricated by Reduced Graphene Oxide/Polyvinyl Butyral Nanofibers for Detecting Cu (II) in Water. Int. J. Anal. Chem..

[84] Liu J., Zhu G., Chen M., Ma X., Yang J. (2016). Fabrication of electrospun ZnO nanofiber-modified electrode for the determination of trace Cd(II). Sens. Actuators B Chem..

[85] Oliveira V.H.B., Rechotnek F., da Silva E.P., de Sousa Marques V., Rubira A.F., Silva R., Lourenço S.A., Muniz E.C. (2020). A sensitive electrochemical sensor for Pb^2+^ ions based on ZnO nanofibers functionalized by L-cysteine. J. Mol. Liq..

[86] Zhu G., Su J., Zhang B., Liu J. (2021). Electrospun amino-containing organosilica gel nanofibers for the ultrasensitive determination of Cu(II). J. Electroanal. Chem..

